# From Aortic Stenosis to Cardiomyopathy: The Hidden Impact of Undiagnosed Acommissural Unicuspid Aortic Valve

**DOI:** 10.1016/j.jaccas.2025.103899

**Published:** 2025-07-03

**Authors:** Muhammad Salman Sabri, Hamza Muhammadzai, Angad Bedi, Eddy Mizrahi, Matthew Collins

**Affiliations:** a Department of Internal Medicine, Jefferson-Abington Hospital, Abington, Pennsylvania, USA; b Department of Cardiology, Thomas Jefferson University Hospital, Philadelphia, Pennsylvania, USA; c Department of Cardiology, Jefferson-Abington Hospital, Abington, Pennsylvania, USA

**Keywords:** aortic stenosis, heart failure, unicuspid aortic valve

## Abstract

Unicuspid aortic valve (UAV) is a rare congenital anomaly, diagnosed in individuals aged 30 to 50. It is associated with aortic stenosis or aortic regurgitation, potentially leading to heart failure. We report a case of a 28-year-old male with an acommissural UAV, aortopathy, critical aortic stenosis, and heart failure with reduced ejection fraction, presenting with shortness of breath. This case is notable for late presentation of an acommissural UAV, which typically manifests in childhood.

## Graphical Abstract

**Figure:**
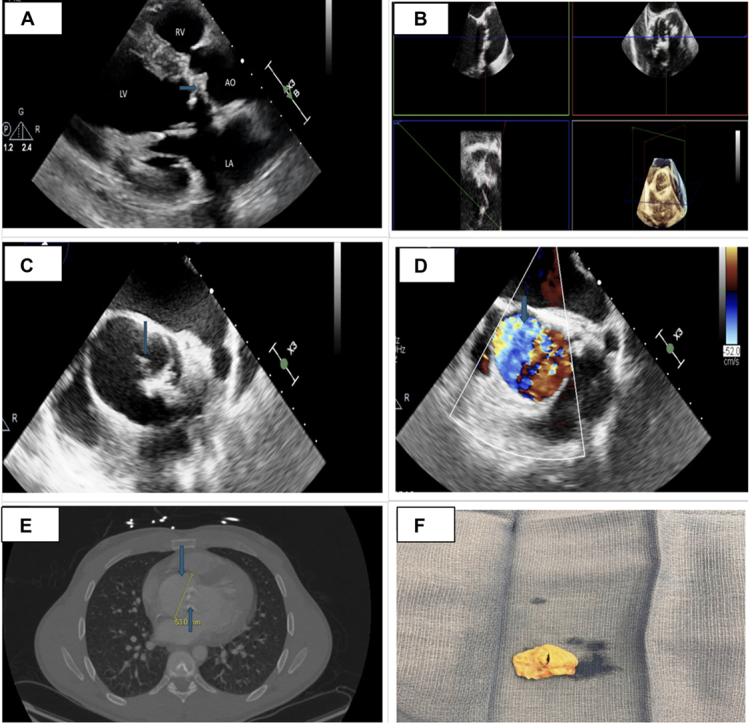

A 28-year-old male, with a past medical history of murmur since childhood, presented with 1-month history of flu-like symptoms followed by exertional dyspnea, and chest discomfort. On examination, his vital signs were stable and had a crescendo-decrescendo murmur at the right upper sternal border with clear lung sounds, and no jugular vein distention or lower extremity edema. The laboratory workup including troponin, N-terminal pro–B-type natriuretic peptide, and D-dimer was unremarkable. Electrocardiogram demonstrated sinus tachycardia, left bundle branch block, and ST-segment depression and T-wave inversion in inferior and lateral leads. transthoracic echocardiography (Figure 1A, Video 1) demonstrated a left ventricular (LV) ejection fraction of 10% to 15%, severe aortic root dilation (6.3 cm), and severe aortic stenosis (mean gradient of 46 mm Hg, aortic valve area of 0.91 cm^2^), with fused aortic valve cusps, suggesting either a unicuspid aortic valve (UAV) or bicuspid aortic valve. Transesophageal echocardiography (Figures 1B to 1D, Video 2, Video 3, Video 4) also demonstrated similar findings with strong suspicion of UAV. A contrast-enhanced computed tomography of the chest and aorta demonstrated calcification of the aortic valve on the gated study, along with dilation of the ascending aortic segment (Figure 1E).

**Take-Home Messages**

• Multimodal imaging is crucial to distinguish UAV from bicuspid aortic valve.
• Surgical intervention is required for accurate diagnosis and treatment of UAV.

**Figure 1:**
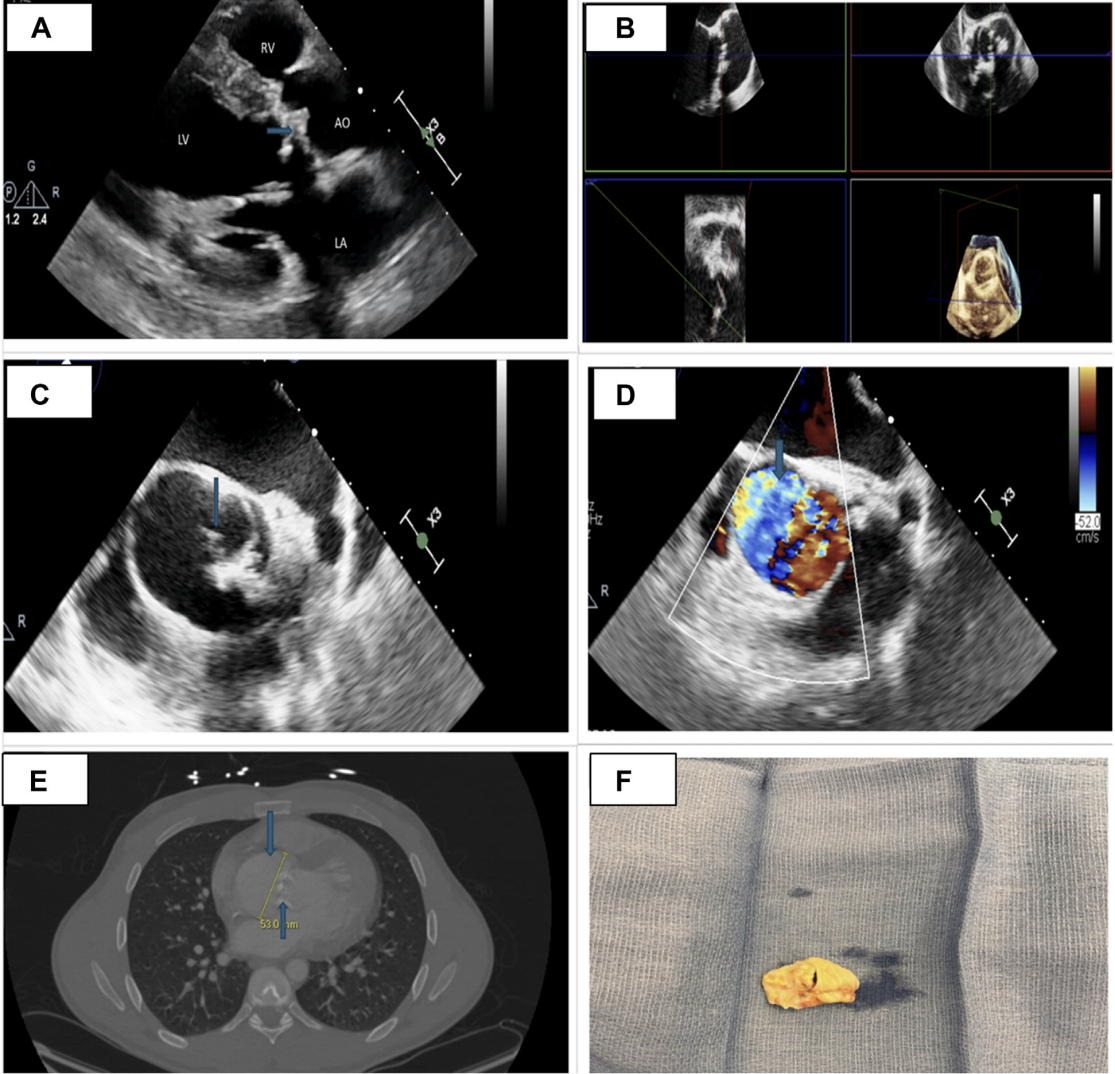
Multimodal Imaging and Gross Pathology (A) The transthoracic echocardiogram shows a dilated left ventricle (LV) with an ejection fraction of 10% to 15%, severe aortic valve stenosis (indicated by the arrow), and dilation of the aortic root. (B to D) The transesophageal echocardiogram confirms severe aortic stenosis with a mean gradient of 34 mm Hg, a unicuspid aortic valve (as shown by the arrow), and aortic root dilation, with no signs of aortic regurgitation. (E) Computed tomography of the chest and aorta (AO) reveals aortic root dilation with a diameter of 53 mm and a calcified aortic valve, as indicated by the arrows. (F) The surgical specimen, highlighting acommissural unicuspid aortic valve with a pinhole-like opening. LA = left atrium; RA = right atrium.

Cardiac catheterization was negative for coronary artery disease, and right heart catheterization showed normal left- and right-sided filling pressures with right atrial pressure of 1 mm Hg, right ventricular pressure of 18/1 mm Hg, pulmonary artery pressure of 18/8 mm Hg, and pulmonary capillary wedge pressure of 5 mm Hg. A respiratory pathogen panel was positive for rhinovirus/enterovirus.

The patient underwent aortic valve replacement with a St. Jude mechanical valve conduit, reimplantation of coronaries, and aortic root replacement. The surgical biopsy specimen of the aortic valve was consistent with acommissural UAV (Figure 1F). He required a pacemaker placement for complete heart block postoperatively. Repeat transthoracic echocardiography showed an improvement in LV ejection fraction to 45% to 50%, with a well-positioned mechanical valve.

UAV is a rare congenital malformation, occurring in 0.02% of the general population (identified via echocardiography) and 4% to 6% of those undergoing aortic valve surgery.^1^ It presents in 2 forms: unicommissural and acommissural.^2^ Patients typically present in their third to fifth decade of life and may have aortic root dilatation, patent ductus arteriosus, aberrant subclavian artery, and coarctation of the aorta.^1^ Symptoms include dyspnea, angina, presyncope, or syncope.^1^^,^^2^ Concentric LV hypertrophy is common in aortic stenosis due to UAV, but chronic cases may develop LV dilatation and eccentric hypertrophy, leading to heart failure.^1^^,^^2^

Although echocardiography is generally the primary diagnostic tool for UAV, supplementary imaging modalities, such as cardiac magnetic resonance imaging, multidetector computed tomography, and in select cases, conventional angiography, can be utilized to assess the severity of stenosis and regurgitation, as well as to evaluate aortic valve morphology and annular size.^1^

The management of UAV usually includes mechanical aortic valve replacement, along with repair of the aortic annulus, root, and ascending aorta if aortopathy is present.^1^^,^^2^ The Ross procedure can be performed in younger patients, by using a pulmonary valve autograft to replace the aortic valve, with great success, low mortality, lower reintervention rates, and no need for anticoagulation.^3^ It was not considered in our patient due to the need for aortic root replacement.

## Funding Support and Author Disclosures

The authors have reported that they have no relationships relevant to the contents of this paper to disclose.

## Appendix

Video 1 The transthoracic echocardiogram shows a dilated left ventricle with an ejection fraction of 15%, left atrial enlargement, and aortic valve stenosis with calcification.

Video 2 The transesophageal echocardiogram reveals a unicuspid aortic valve with severe valve narrowing and calcification.

Video 3 The transesophageal echocardiogram with color Doppler demonstrates unicuspid aortic valve but no evidence of significant aortic regurgitation.

Video 4 The transesophageal echocardiogram shows a small, pinhole-sized opening, indicative of acommissural unicuspid aortic valve.
